# Supplementary figures and images for: Protein signatures linking history of miscarriages and metabolic syndrome: a proteomic study among North Indian women
Source: PeerJ. 2019 Feb 14;7:e6321. doi: 10.7717/peerj.6321 (PMC6378092; doi:10.7717/peerj.6321)

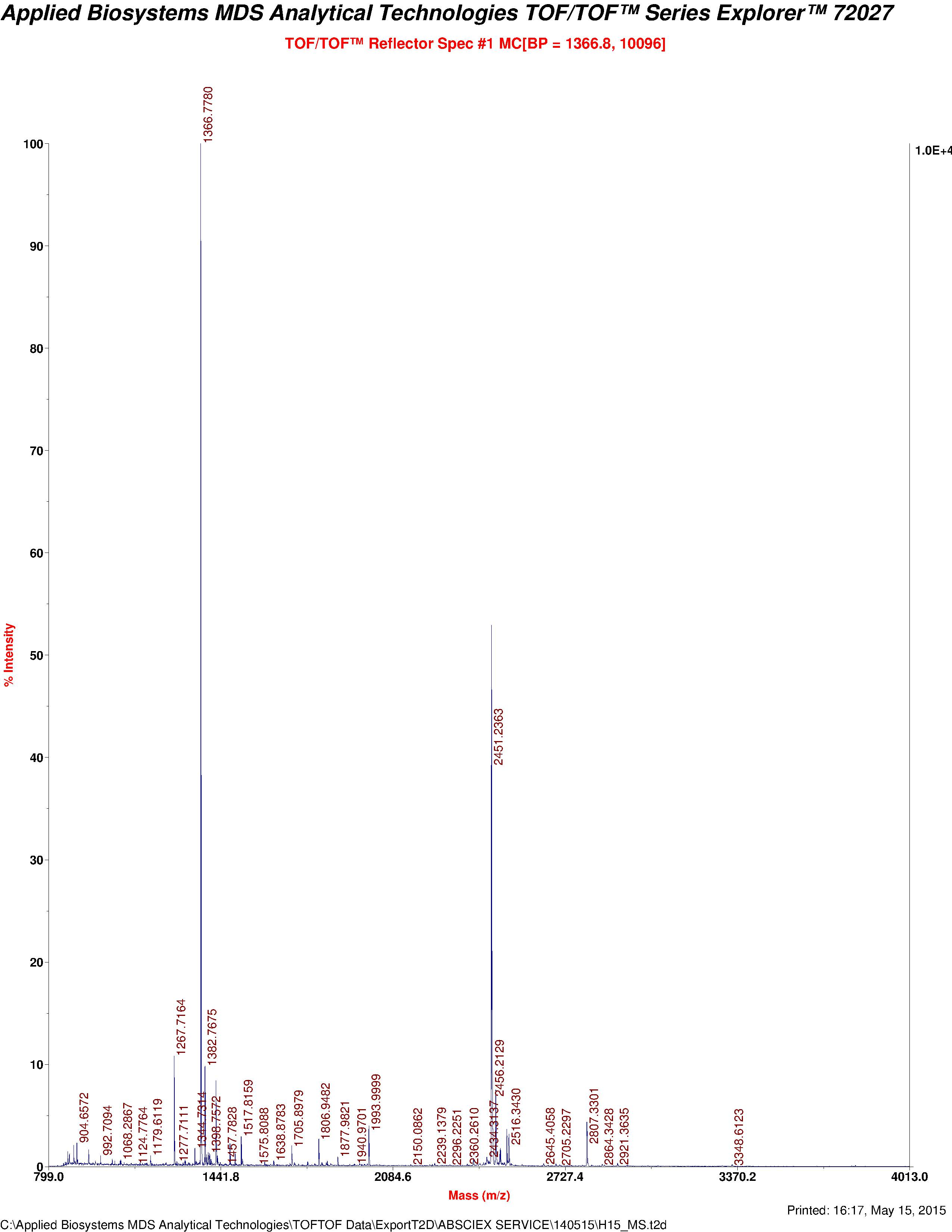

Supplement: Supplemental Information 1 [file peerj-07-6321-s001.png]

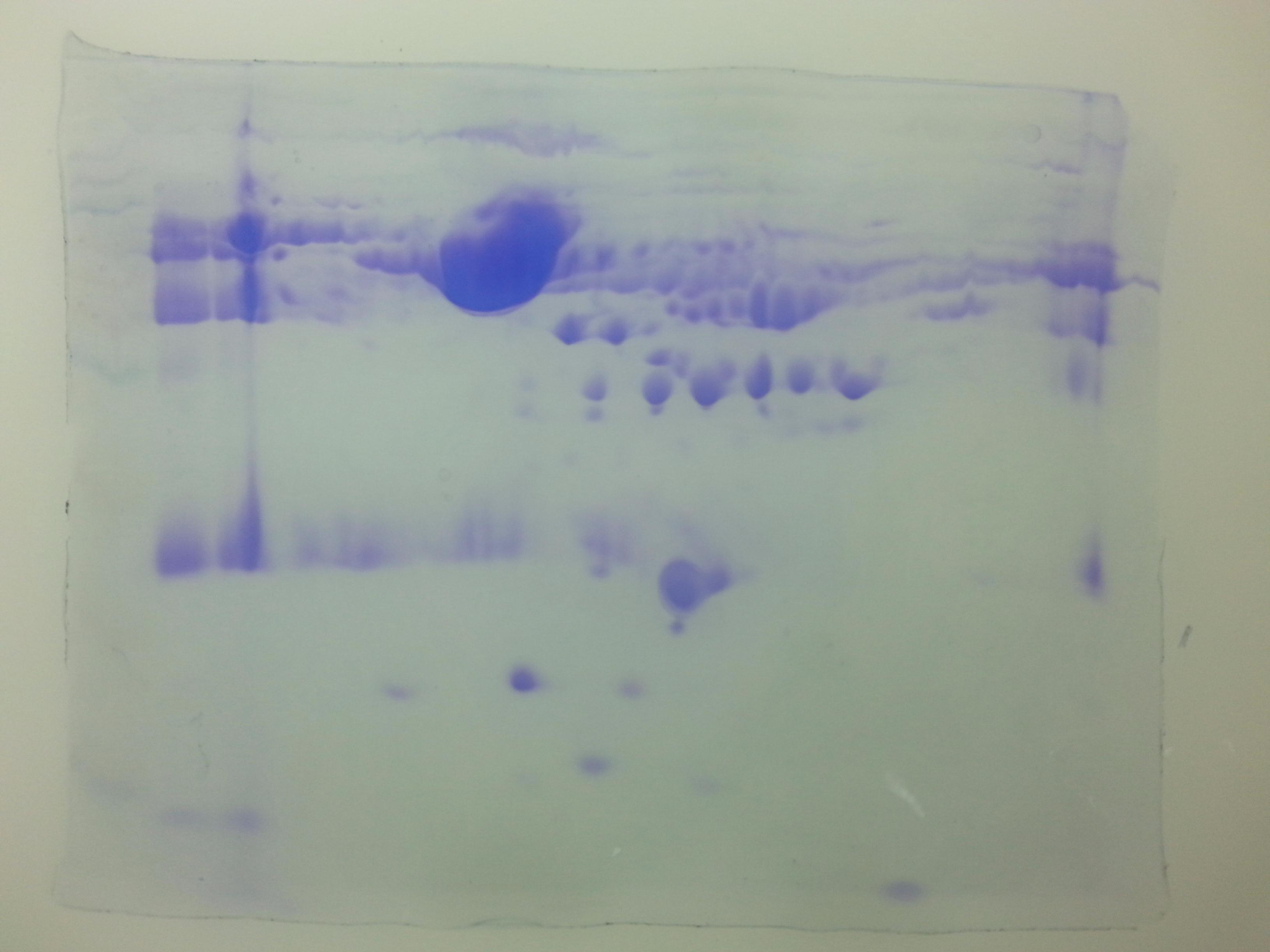

Supplement: Supplemental Information 10 [file peerj-07-6321-s010.jpg]

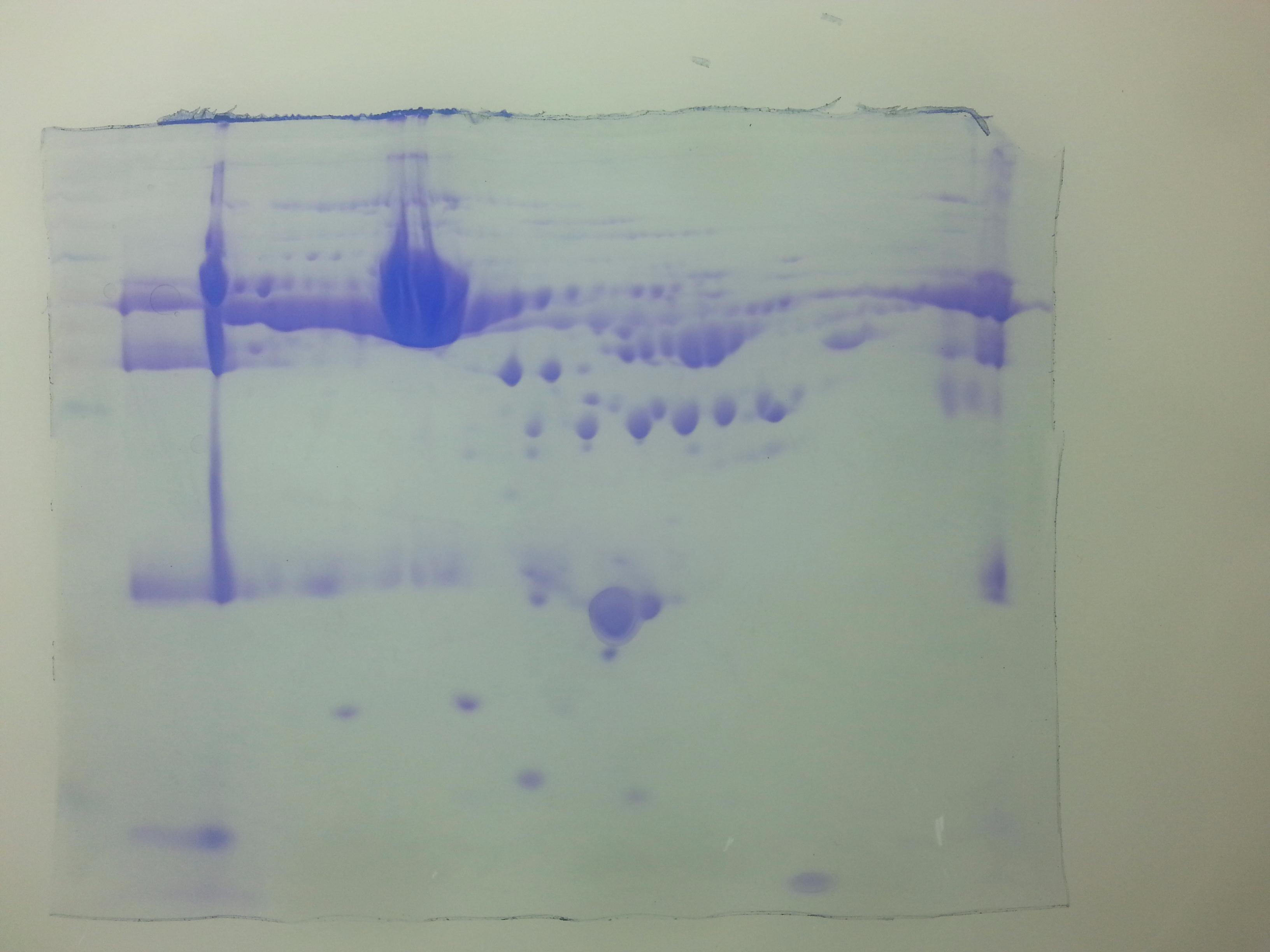

Supplement: Supplemental Information 11 [file peerj-07-6321-s011.jpg]

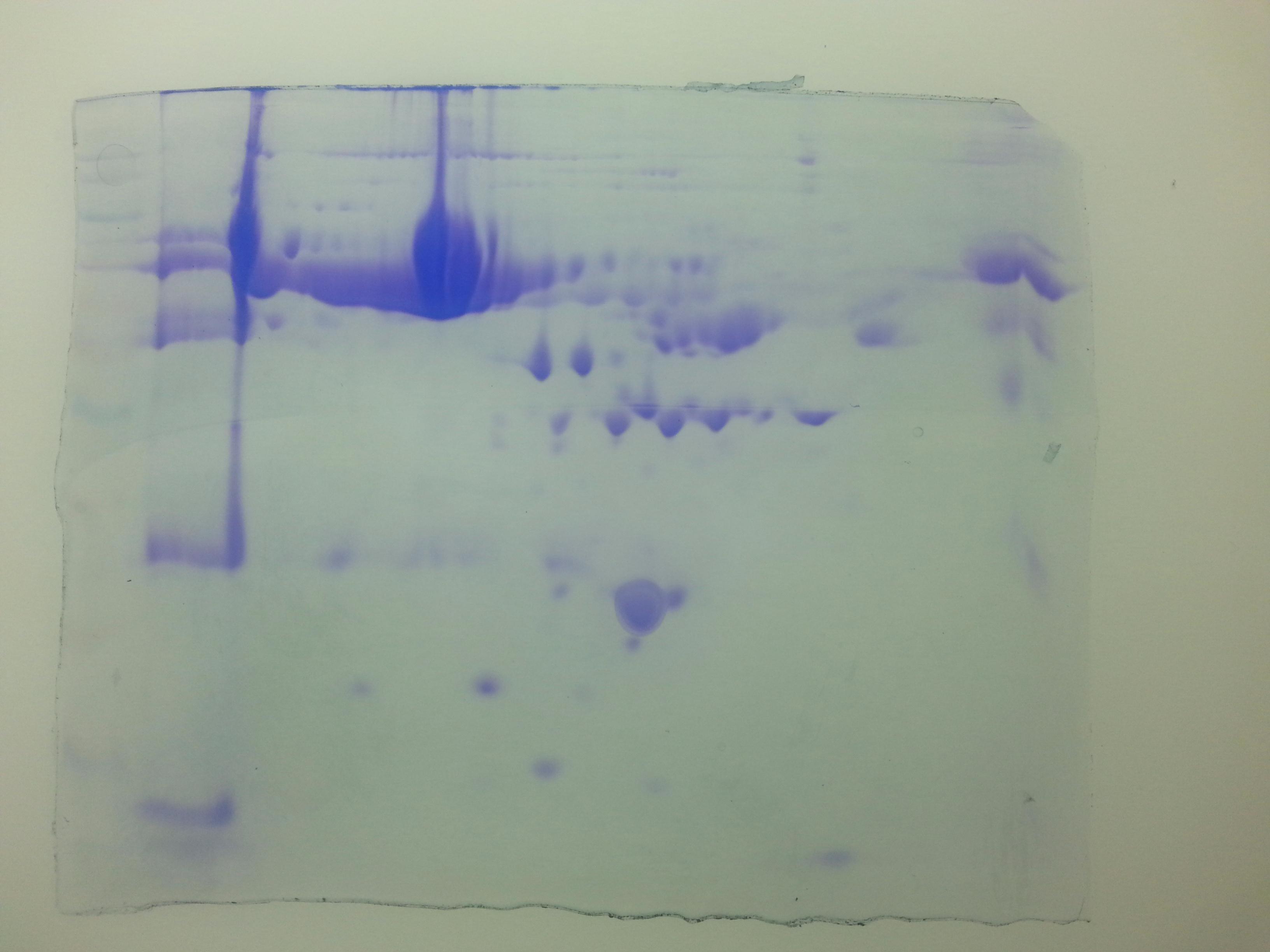

Supplement: Supplemental Information 12 [file peerj-07-6321-s012.jpg]

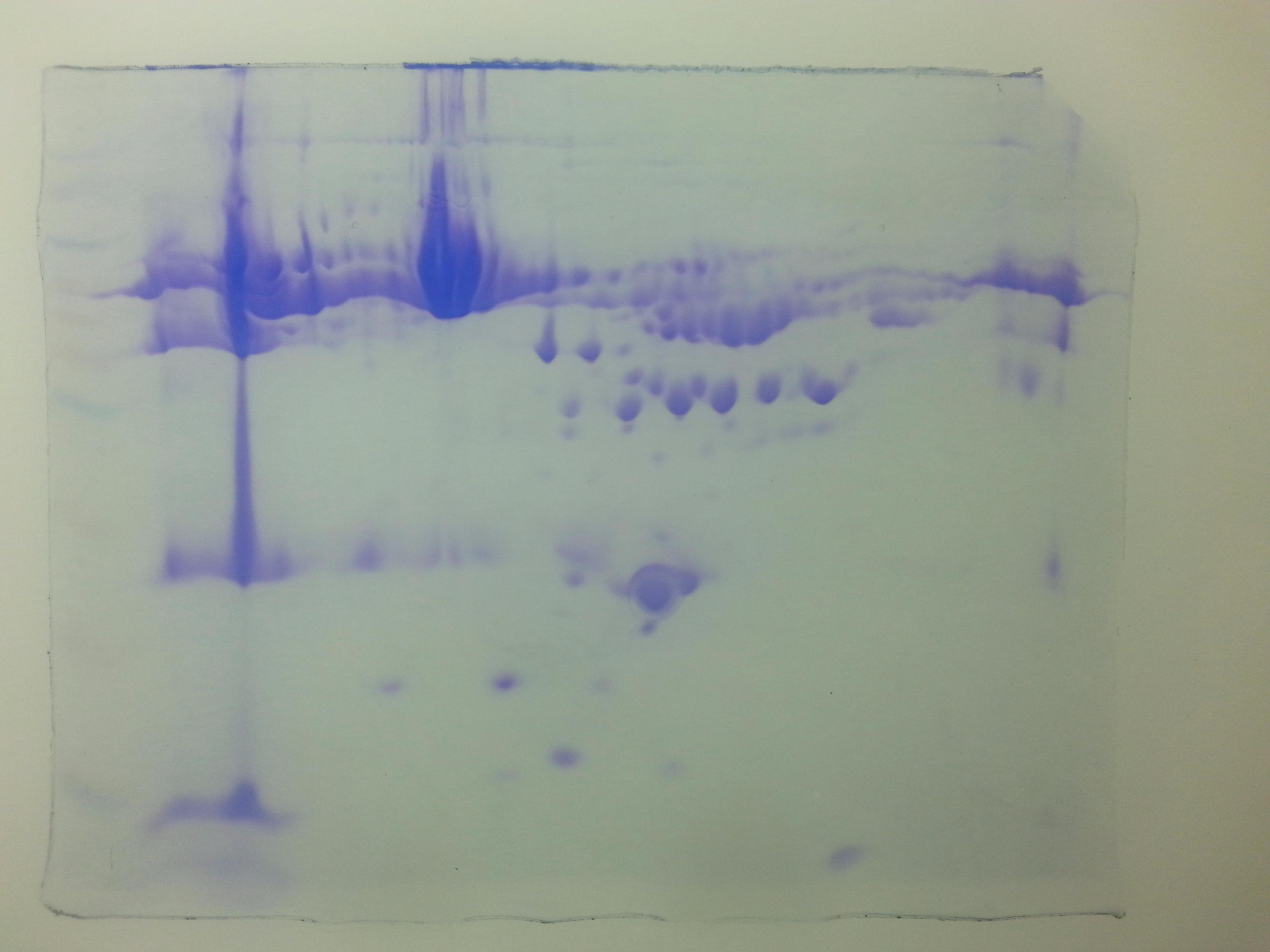

Supplement: Supplemental Information 13 [file peerj-07-6321-s013.jpg]
