# Supplementary material for: Protein signatures linking history of miscarriages and metabolic syndrome: a proteomic study among North Indian women
Source: PeerJ. 2019 Feb 14;7:e6321. doi: 10.7717/peerj.6321 (PMC6378092; doi:10.7717/peerj.6321)
Supplement: Supplemental Information 2 [file peerj-07-6321-s002.doc]

**Table S1: Demographic and biochemical characteristics of the study groups**

| **Variables** | **Group A** | **Group B** | **Group C** | **Group D** | **p-value** |
| --- | --- | --- | --- | --- | --- |
| **Present Age (years)** | 39.4±3.4 | 39.4±3.3 | 38.8±3.9 | 38.2±3.2 | 0.62 |
| **Age at Menarche (years)** | 14.6±1.0 | 14.6±1.6 | 15.2±2.0 | 15.0±1.6 | 0.55 |
| **Age at Marriage (years)** | 15.8±2.3 | 16.5±2.5 | 15.9±2.5 | 15.9±2.3 | 0.74 |
| **Age at first conception (years)** | 18.3±2.7 | 19.4±3.3 | 15.2±2.0 | 19.3±2.8 | 0.03 |
| **Age at last conception (years)** | 26.5±4.4 | 28.3±5.0 | 28.4±5.6 | 29.5±5.2 | 0.30 |
| **Weight (kg)** | 60.4±8.6 | 50.8±9.6 | 56.4±9.4 | 50.2±7.9 | 0.001 |
| **Height (cm)** | 153.4±4.5 | 155.9±5.2 | 155.4±4.8 | 154.9±4.9 | 0.42 |
| **BMI (kg/m^2^)** | 25.5±3.2 | 20.9±4.1 | 23.3±3.4 | 20.9±3.2 | <0.001 |
| **SBP (mm Hg)** | 117.4±15.3 | 119.6±17.2 | 129.1±21.9 | 113.0±12.4 | 0.02 |
| **DBP (mm Hg)** | 90.5±14.4 | 80.0±6.8 | 92.9±22.1 | 79.3±14.9 | 0.009 |
| **WC (cm)** | 88.9±9.8 | 77.6±11.7 | 87.7±8.2 | 76.9±8.8 | <0.001 |
| **WHR** | 0.89±0.04 | 0.85±0.09 | 0.91±0.05 | 0.84±0.06 | 0.002 |
| **Fasting Glucose (mg/dL)** | 102.2±56.2 | 76.0±15.8 | 86.4±16.5 | 74.2±13.1 | 0.02 |
| **TC (mg/dL)** | 199.6±72.0 | 166.3±38.9 | 204.3±56.6 | 153.4±28.1 | 0.005 |
| **TG (mg/dL)** | 158.4±71.5 | 99.3±42.0 | 150.6±61.3 | 97.2±44.4 | <0.001 |
| **HDL (mg/dL)** | 44.8±15.2 | 55.3±13.9 | 47.2±15.2 | 53.2±12.8 | 0.07 |
| **LDL (mg/dL)** | 123.1±72.9 | 91.1±34.5 | 125.9±54.3 | 83.9±32.4 | 0.01 |

BMI – body mass index, SBP – systolic blood pressure, DBP – diastolic blood pressure, WC – waist circumference, WHR – waist-hip ratio, TC – total cholesterol, TG – triglyceride, HDL – high density lipoprotein, LDL – low density lipoprotein
