# Supplementary material for: Protein signatures linking history of miscarriages and metabolic syndrome: a proteomic study among North Indian women
Source: PeerJ. 2019 Feb 14;7:e6321. doi: 10.7717/peerj.6321 (PMC6378092; doi:10.7717/peerj.6321)
Supplement: Supplemental Information 14 [file peerj-07-6321-s014.doc]

T-TEST GROUPS=VAR00001(1 2)

  /MISSING=ANALYSIS

  /VARIABLES=VAR00002

  /CRITERIA=CI(.9500).

**T-Test**

| **Notes** | | |
| --- | --- | --- |
| Output Created | | 17-Oct-2018 12:10:05 |
| Comments | |  |
| Input | Active Dataset | DataSet0 |
|  | Filter | <none> |
|  | Weight | <none> |
|  | Split File | <none> |
|  | N of Rows in Working Data File | 40 |
| Missing Value Handling | Definition of Missing | User defined missing values are treated as missing. |
|  | Cases Used | Statistics for each analysis are based on the cases with no missing or out-of-range data for any variable in the analysis. |
| Syntax | | T-TEST GROUPS=VAR00001(1 2)  /MISSING=ANALYSIS  /VARIABLES=VAR00002  /CRITERIA=CI(.9500). |
| Resources | Processor Time | 00:00:00.031 |
|  | Elapsed Time | 00:00:00.047 |

[DataSet0]

| **Group Statistics** | | | | | |
| --- | --- | --- | --- | --- | --- |
|  | VAR00001 | N | Mean | Std. Deviation | Std. Error Mean |
| VAR00002 | 1 | 20 | .8340 | .07493 | .01676 |
|  | 2 | 20 | .3332 | .08627 | .01929 |

| **Independent Samples Test** | | | | | | | | | | |
| --- | --- | --- | --- | --- | --- | --- | --- | --- | --- | --- |
|  |  | Levene's Test for Equality of Variances | | t-test for Equality of Means | | | | | | |
|  |  | F | Sig. | t | df | Sig. (2-tailed) | Mean Difference | Std. Error Difference | 95% Confidence Interval of the Difference | |
|  |  |  |  |  |  |  |  |  | Lower | Upper |
| VAR00002 | Equal variances assumed | 1.469 | .233 | 19.600 | 38 | .000 | .50079 | .02555 | .44906 | .55251 |
|  | Equal variances not assumed |  |  | 19.600 | 37.270 | .000 | .50079 | .02555 | .44903 | .55255 |

T-TEST GROUPS=VAR00001(1 2)

  /MISSING=ANALYSIS

  /VARIABLES=VAR00002

  /CRITERIA=CI(.9500).

**T-Test**

| **Notes** | | |
| --- | --- | --- |
| Output Created | | 17-Oct-2018 12:10:49 |
| Comments | |  |
| Input | Active Dataset | DataSet0 |
|  | Filter | <none> |
|  | Weight | <none> |
|  | Split File | <none> |
|  | N of Rows in Working Data File | 40 |
| Missing Value Handling | Definition of Missing | User defined missing values are treated as missing. |
|  | Cases Used | Statistics for each analysis are based on the cases with no missing or out-of-range data for any variable in the analysis. |
| Syntax | | T-TEST GROUPS=VAR00001(1 2)  /MISSING=ANALYSIS  /VARIABLES=VAR00002  /CRITERIA=CI(.9500). |
| Resources | Processor Time | 00:00:00.047 |
|  | Elapsed Time | 00:00:00.062 |

[DataSet0]

| **Group Statistics** | | | | | |
| --- | --- | --- | --- | --- | --- |
|  | VAR00001 | N | Mean | Std. Deviation | Std. Error Mean |
| VAR00002 | 1 | 20 | .8340 | .07493 | .01676 |
|  | 2 | 20 | .3731 | .07501 | .01677 |

| **Independent Samples Test** | | | | | | | | | | |
| --- | --- | --- | --- | --- | --- | --- | --- | --- | --- | --- |
|  |  | Levene's Test for Equality of Variances | | t-test for Equality of Means | | | | | | |
|  |  | F | Sig. | t | df | Sig. (2-tailed) | Mean Difference | Std. Error Difference | 95% Confidence Interval of the Difference | |
|  |  |  |  |  |  |  |  |  | Lower | Upper |
| VAR00002 | Equal variances assumed | .000 | .992 | 19.440 | 38 | .000 | .46089 | .02371 | .41289 | .50888 |
|  | Equal variances not assumed |  |  | 19.440 | 38.000 | .000 | .46089 | .02371 | .41289 | .50888 |

T-TEST GROUPS=VAR00001(1 2)

  /MISSING=ANALYSIS

  /VARIABLES=VAR00002

  /CRITERIA=CI(.9500).

**T-Test**

| **Notes** | | |
| --- | --- | --- |
| Output Created | | 17-Oct-2018 12:11:26 |
| Comments | |  |
| Input | Active Dataset | DataSet0 |
|  | Filter | <none> |
|  | Weight | <none> |
|  | Split File | <none> |
|  | N of Rows in Working Data File | 40 |
| Missing Value Handling | Definition of Missing | User defined missing values are treated as missing. |
|  | Cases Used | Statistics for each analysis are based on the cases with no missing or out-of-range data for any variable in the analysis. |
| Syntax | | T-TEST GROUPS=VAR00001(1 2)  /MISSING=ANALYSIS  /VARIABLES=VAR00002  /CRITERIA=CI(.9500). |
| Resources | Processor Time | 00:00:00.000 |
|  | Elapsed Time | 00:00:00.000 |

[DataSet0]

| **Group Statistics** | | | | | |
| --- | --- | --- | --- | --- | --- |
|  | VAR00001 | N | Mean | Std. Deviation | Std. Error Mean |
| VAR00002 | 1 | 20 | .8340 | .07493 | .01676 |
|  | 2 | 20 | .2034 | .04750 | .01062 |

| **Independent Samples Test** | | | | | | | | | | |
| --- | --- | --- | --- | --- | --- | --- | --- | --- | --- | --- |
|  |  | Levene's Test for Equality of Variances | | t-test for Equality of Means | | | | | | |
|  |  | F | Sig. | t | df | Sig. (2-tailed) | Mean Difference | Std. Error Difference | 95% Confidence Interval of the Difference | |
|  |  |  |  |  |  |  |  |  | Lower | Upper |
| VAR00002 | Equal variances assumed | .997 | .324 | 31.787 | 38 | .000 | .63061 | .01984 | .59045 | .67077 |
|  | Equal variances not assumed |  |  | 31.787 | 32.149 | .000 | .63061 | .01984 | .59021 | .67101 |
